# Cesarean delivery rate and staffing levels of the maternity unit

**DOI:** 10.1371/journal.pone.0207379

**Published:** 2018-11-28

**Authors:** Saad Zbiri, Patrick Rozenberg, François Goffinet, Carine Milcent

**Affiliations:** 1 EA 7285, Versailles Saint Quentin University, Montigny-le-Bretonneux, France; 2 Department of Obstetrics and Gynecology, Poissy Saint-Germain Hospital, Poissy, France; 3 Port Royal Maternity Unit, Cochin Hospital, Assistance Publique des Hôpitaux de Paris, DHU Risks and Pregnancy, Paris, France; 4 INSERM U1153, Obstetrical, Perinatal and Pediatric Epidemiology Research Team (EPOPé), Research Center for Epidemiology and Biostatistics Sorbonne Paris Cité (CRESS), Paris Descartes University, Paris, France; 5 Paris School of Economics (PSE), Paris, France; 6 French National Center for Scientific Research (CNRS), Paris, France; PreTel, UNITED STATES

## Abstract

**Objective:**

To investigate whether staffing levels of maternity units affect prelabor urgent, elective, and intrapartum cesarean delivery rates.

**Methods:**

This population-based retrospective cohort study covers the deliveries of the 11 hospitals of a French perinatal network in 2008–2014 (N = 102 236). The independent variables were women’s demographic and medical characteristics as well as the type, organization, and staffing levels for obstetricians, anesthesiologists, and midwives of each maternity unit. Bivariate and multivariate analyses were conducted with multilevel logistic models.

**Results:**

Overall, 23.9% of the women had cesarean deliveries (2.4% urgent before labor, 10% elective, and 11.5% intrapartum). Independently of individual- and hospital-level factors, the level of obstetricians, measured by the number of full-time equivalent persons (i.e., 35 working hours per week) per 100 deliveries, was negatively associated with intrapartum cesarean delivery (adjusted odds ratio, aOR 0.55, 95% confidence interval, CI 0.36–0.83, *P*-value = 0.005), and the level of midwives negatively associated with elective cesarean delivery (aOR 0.79, 95% CI 0.69–0.90, *P*-value < 0.001). Accordingly, a 10% increase in obstetrician and midwife staff levels, respectively, would have been associated with a decrease in the likelihood of intrapartum cesarean delivery by 2.5 percentage points and that of elective cesarean delivery by 3.4 percentage points. These changes represent decreases in intrapartum and elective cesarean delivery rates of 19% (from 13.1% to 10.6%) and 33% (from 10.3% to 6.9%), respectively.

**Conclusion:**

Staffing levels of maternity units affect the use of cesarean deliveries. High staffing levels for obstetricians and midwives are associated with lower cesarean rates.

## Introduction

Cesarean delivery rates have risen steadily in recent decades. Between 1990 and 2014, the absolute increases in these rates were 19% in Latin America, 15% in Asia, 14% in Europe, and 10% in North America [1–3]. This increase, which has affected especially high-income countries with widespread access to medical services [2, 4, 5], has taken place despite the lack of evidence that it provides additional benefits for either the mother or the baby [6–8]. Currently cesarean rates exceed 50% in Brazil and are slightly lower in China; they are around 30% in the USA and Germany and 25% in the UK [4, 9, 10]. In France, the cesarean rate rose from 15% in 1995 to 20% in 2003, but remained stable around 21% till 2016 [11, 12]. It nonetheless continues to show a significant degree of unexplained clinical variation across hospitals [12].

Cesarean deliveries may lead to significant adverse consequences for maternal and child health. Maternal risks include postpartum infection, venous thromboembolism and anesthesia-related complications [13], and for future pregnancies, fertility disorders and especially placenta previa and/or accreta [14, 15]. Some of these may lead to maternal death [16]. Child health disorders associated with cesareans include neonatal respiratory distress [17], and childhood asthma and obesity [18, 19]. Finally, they impair the health system by increasing hospital costs [20, 21]. For all these reasons, reducing cesarean rates is a major public-health objective.

The determinants of cesarean deliveries are interrelated, complex, and may differ from one country to another. Because clinical indications are unlikely to explain more than a portion of all cesarean deliveries [22, 23], the involvement of nonclinical factors should also be studied. Individual preferences and characteristics of women affect cesarean use [24–26], as do physicians’ attitudes and incentives [27–29]. Factors related to the maternity unit are also important. Many studies have shown the heterogeneity of cesarean delivery practices across hospitals, varying according to their ownership status [30–32], facility level [33], teaching status [34], and organization [35–37]. However, only a few analyses have examined the impact of hospital staffing on cesarean births [38–40], even though several studies have previously shown that some specific characteristics of professionals, such as physician’s gender or fear of malpractice litigation, are explanatory factors for the decision to perform a cesarean delivery [41–43].

The objective of this study was to assess the effect of maternity unit staffing levels for healthcare professionals on cesarean rates, while taking characteristics of women and maternity units into account. We hypothesized that if staffing levels affect cesarean delivery rates, the increase of cesarean delivery rates over recent decades might be attributable in part to changes in these levels, given that hospitals have gradually been induced to control their staff levels to reduce their costs [44].

## Materials and methods

We conducted a population-based retrospective cohort study of deliveries in the perinatal network covering the district of Yvelines (west of Paris) in 2008 through 2014. The network comprises 11 maternity units including two hospitals affiliated to a university (Université Versailles-Saint Quentin). Data were extracted from two French datasets. The first, CoNaissance 78, is a program that monitors maternal and perinatal morbidity and mortality. It includes all deliveries in the district. This dataset contains demographic characteristics and medical information about the pregnancy, the delivery, and maternal, fetal, and neonatal health. Data are continuously recorded from the first health certificate of infants born in network maternity units; another health certificate reports additional data such as severe maternal morbidity, and a stillbirth certificate for all fetal deaths and medical terminations of pregnancy from 22 weeks of gestation. These certificates are completed by midwives and physicians and then collected and recorded; regular quality control takes place. The missing data rates were < 3% for all variables in this study. The second dataset, the Annual Statistics for French Hospitals, provides detailed information on the characteristics and location of all hospitals in France, compiled by the Ministry of Health, from information the hospitals provide. The two datasets were linked by using the name of the hospital of delivery and its identification number. The National Committee for Data Protection (Commission Nationale de l'Informatique et des Libertés, registration number 1295794) approved the study, which was conducted in accordance with French legislation. Because the study was about standard care and because the dataset contained no information that could enable patient identification, the study was exempt from informed consent requirements and from Institutional Review Board approval.

Our variables were selected according to the previous literature on cesarean deliveries in high-income countries. Previous studies have shown that demographic [45, 46], medical [47, 48], and hospital factors may affect the use of cesarean deliveries [30–37]. The demographic characteristics included were maternal age and parity (nulliparous or parous). The following medical characteristics were considered: previous cesarean, medical risk condition, plurality (singleton or multiple), preterm delivery (defined by a gestational age < 37 weeks), fetal presentation (cephalic or breech/transverse), induced labor, and birth weight (< 2500 grams, 2500 to 4000 grams, or > 4000 grams). A medical risk condition was defined by any of the following maternal or fetal conditions and co-morbidities: diabetes, hypertension, preeclampsia (including HELLP syndrome and eclampsia), fetal growth restriction, placental bleeding (including abruptio placentae, placenta previa, and placenta accreta), or other pregnancy conditions (such as obesity, infection, or congenital anomaly). Hospital (maternity unit) information used was type, organization, and staffing. Type included ownership status (public or private), university status (non-teaching or teaching), and level of neonatal care (no neonatology unit, neonatology unit, or neonatal intensive care unit). Maternity unit organizational factors considered were: day of delivery (working day or weekend/holiday), obstetrician availability (present 24 hours per day and 7 days per week in the unit or on-call but not necessarily present), and size of unit, measured by the number of annual deliveries (< 1000, 1000 to 1999, or ≥ 2000). Finally, staffing of the maternity unit included the number of obstetricians, of anesthesiologists, and of midwives.

Hospitals reported their staffing in terms of full-time equivalents (FTEs) according to the available information on working hours. A FTE corresponds to 35 working hours per week. We used the average annual FTE of each staff category, which took into account the possible variations that may exist across weeks, and was thus comparable between hospitals. We considered all professionals of the maternity unit involved in decisions about cesarean delivery (i.e., obstetricians, anesthesiologists, and midwives), as they were not assigned to a particular ward of the maternity unit (e.g., labor ward, outpatient clinic, inpatient unit), and as the cesarean delivery decision may be made at different times during pregnancy and delivery. In particular, French maternity units usually provide prenatal care for women scheduled to deliver there. The numbers of FTEs were related to the total number of deliveries per year and expressed as numbers of FTEs per 100 deliveries. Because private-practice physicians in private hospitals are self-employed, their working hours were not known and they were reported only as numbers of individuals. To consider the actual practice rates of those part-time private-practice physicians, we used the assumption that each spent 50% of his or her time at the hospital. This standard assumption has been used in previous studies [49]. Nonetheless, we conducted a sensitivity analysis to test the robustness of our results, applying two more extreme assumptions (25% and 75%).

Mode of delivery was the variable of interest. Because many factors associated with cesarean delivery depend on the timing of delivery (before or during labor) or the degree of urgency or both, we conducted three distinct analyses to obtain the most appropriate comparison groups for our variable of interest: (1) prelabor urgent cesarean deliveries (such as those performed for severe fetal growth restriction or abruptio placentae) versus all other deliveries including elective and intrapartum cesarean and vaginal deliveries; (2) planned elective cesarean deliveries versus all deliveries with a planned vaginal delivery, including both intrapartum cesarean and vaginal deliveries; and (3) intrapartum cesarean deliveries versus all vaginal deliveries [50].

The structure of our data was hierarchical, with women (first level) nested within maternity units (second level). We therefore used multilevel logistic regression models with hospital-specific random intercepts and estimated the robust variance that accounted for the dependence between observations. These models took differences in unmeasured hospital-level characteristics into account and considered the lack of independence of observations within each hospital by assuming that women from the same maternity unit were more likely to deliver in the same way than women from different units [51]. Both bivariate (adjusted for the trend) and multivariate analyses were conducted. For the bivariate analysis, we calculated odds ratios (ORs) with their 95% confidence intervals (CIs). Because many variables were correlated, all factors, regardless of their association with cesarean delivery in the bivariate analysis, were considered for the multivariate analysis, and the final models included all variables. All models included a trend to capture the heterogeneity in time and to examine the course of cesarean rates over the study period. Results are reported as adjusted ORs (aORs) with their 95% CIs. In all analyses, a two-sided *P*-value < 0.050 was considered significant. When any staffing level was significantly associated with cesarean delivery, we estimated the percentage change in the probability of cesarean delivery predicted to be associated with a percentage change in this level (elasticity) [52]. A sensitivity analysis used hospital fixed effects models to consider possible correlations between the characteristics of maternity units not explicitly taken into account in the final models and any staff variable. All statistical analyses used Stata 13.1 software (Stata Corporation, College Station, TX, USA) [53].

## Results

The analysis included 102 236 live deliveries during the study period. Of these, 77 766 had vaginal deliveries (spontaneous or instrumental) and 24 470 cesarean deliveries. The percentage of cesarean deliveries was 23.9% (95% CI 23.7–24.2). Overall, 11 719 (47.9%) were intrapartum, 10 243 (41.9%) elective, and 2588 (10.2%) prelabor urgent. Table 1 presents the characteristics of the study population, including the mean of the staffing level variables.

**Table 1:** Distribution of characteristics of the study population.

	Deliveries (N = 102 236)
**Women’s characteristics**	
Maternal age (years), mean ± SD	30.73 ± 5.14
Nulliparous, n (%)	44 058 (43.1)
Previous cesarean, n (%)	10 564 (10.3)
Medical risk condition, n (%)	13 523 (13.2)
Multiple pregnancy, n (%)	1493 (1.5)
Preterm delivery, n (%)	5149 (5.0)
Breech/transverse presentation, n (%)	4184 (4.1)
Induced labor, n (%)	22 064 (21.6)
Birth weight (grams), n (%)	
< 2500	5606 (5.5)
2500–4000	89 403 (87.4)
> 4000	7227 (7.1)
**Maternity unit characteristics**	
Private, n (%)	34 396 (33.6)
Teaching, n (%)	38 329 (37.5)
Level of care, n (%)	
No neonatology unit	19 541 (19.1)
Neonatology unit	40 524 (39.6)
Neonatal intensive care unit	42 171 (41.3)
Weekend/holiday delivery	27 006 (26.4)
On-call obstetrician outside the unit, n (%)	9293 (9.1)
Size (deliveries/year), n (%)	
< 1000	11 861 (11.6)
1000–1999	32 571 (31.9)
≥ 2000	57 804 (56.5)
Obstetricians (FTEs/100 deliveries), mean ± SD	0.49 ± 0.14
Anesthesiologists (FTEs/100 deliveries), mean ± SD	0.56 ± 0.21
Midwives (FTEs/100 deliveries), mean ± SD	1.55 ± 0.45

FTEs, full-time equivalents.

According to the bivariate analysis, staffing levels of the maternity unit were associated with cesarean deliveries (Table 2). Notably, the number of FTE obstetricians per 100 deliveries was negatively associated with intrapartum cesarean delivery (OR 0.54, 95% CI 0.40–0.71, *P*-value < 0.001). The number of FTE anesthesiologists and that of FTE midwives per 100 deliveries were not associated with cesarean deliveries. We checked that the number of deliveries was not significantly correlated with the cesarean delivery rate (S1 Table).

**Table 2:** Bivariate analysis of factors associated with cesarean deliveries. Multilevel logistic regression models with hospital random effects.

	OR [95% CI]		
	Urgent cesarean ^a^	Elective cesarean ^b^	Intrapartum cesarean ^c^
	(n = 2508/102 236)	(n = 10 243/99 728)	(n = 11 719/89 485)
**Women’s characteristics**			
Maternal age (years)	1.04 (1.03–1.05)	1.08 (1.07–1.09)	1.00 (0.99–1.01)
Nulliparous	0.96 (0.75–1.22)	0.41 (0.33–0.51)	2.67 (2.41–2.97)
Previous cesarean	3.95 (3.25–4.81)	18.44 (15.99–21.27)	3.71 (3.45–3.99)
Medical risk condition	3.61 (2.92–4.47)	1.57 (1.42–1.74)	1.59 (1.44–1.75)
Multiple pregnancy	4.58 (3.24–6.46)	5.18 (4.47–6.01)	3.98 (3.35–4.73)
Preterm delivery	11.46 (9.27–14.17)	1.63 (1.10–2.40)	2.23 (1.82–2.73)
Breech/transverse presentation	7.66 (6.20–9.46)	19.95 (16.58–23.99)	11.37 (8.70–14.86)
Induced labor	-	-	2.22 (2.06–2.39)
Birth weight (grams)			
< 2500	9.34 (7.79–11.20)	2.06 (1.63–2.59)	2.71 (2.27–3.24)
2500–4000	1	1	1
> 4000	0.82 (0.62–1.09)	1.34 (1.01–1.78)	1.70 (1.60–1.81)
**Maternity unit characteristics**			
Private	1.10 (0.84–1.44)	1.82 (1.28–2.58)	1.06 (0.85–1.32)
Teaching	1.04 (0.65–1.65)	0.80 (0.47–1.37)	1.12 (0.96–1.31)
Level of care			
No neonatology unit	1	1	1
Neonatology unit	1.32 (0.97–1.80)	1.25 (1.12–1.38)	1.05 (0.97–1.14)
Neonatal intensive care unit	1.38 (0.93–2.04)	1.00 (0.63–1.59)	1.12 (0.91–1.37)
Weekend/holiday delivery	0.91 (0.82–1.01)	0.12 (0.09–0.15)	0.93 (0.89–0.97)
On-call obstetrician outside the unit	1.06 (0.73–1.53)	1.16 (1.03–1.30)	1.11 (0.95–1.29)
Size (deliveries/year)			
< 1000	1.02 (0.82–1.28)	0.99 (0.93–1.04)	0.87 (0.78–0.98)
1000–1999	1	1	1
≥ 2000	1.19 (1.07–1.33)	1.02 (0.93–1.12)	1.00 (0.91–1.10)
Obstetricians (FTEs/100 deliveries)	1.11 (0.62–1.99)	0.83 (0.62–1.10)	0.54 (0.40–0.71)
Anesthesiologists (FTEs/100 deliveries)	1.42 (0.64–3.18)	1.07 (0.69–1.66)	1.17 (0.74–1.84)
Midwives (FTEs/100 deliveries)	1.16 (0.81–1.66)	0.84 (0.64–1.09)	1.06 (0.80–1.41)

OR, odds ratio; CI, confidence interval; FTEs, full-time equivalents.

^a^ Urgent cesareans were compared with all other deliveries (elective cesareans, intrapartum cesareans, and vaginal deliveries).

^b^ Elective cesareans were compared with all deliveries with a trial of labor (intrapartum cesareans and vaginal deliveries).

^c^ Intrapartum cesareans were compared with all vaginal deliveries.

After adjustment for all factors in the multivariate models, maternity unit staff levels were associated with cesarean deliveries (Table 3). As expected, well-known individual- and hospital-level characteristics, used as control variables, had significant effects on the mode of delivery. The independent significant variables associated with prelabor urgent cesarean delivery were maternal age, nulliparity, previous cesarean, medical risk condition, multiple pregnancy, preterm delivery, breech/transverse presentation, birth weight, and size of unit. Those associated with elective cesarean delivery were maternal age, previous cesarean, medical risk condition, preterm delivery, breech/transverse presentation, birth weight, private status, level of neonatal care, weekend/holiday delivery, and size of unit. Intrapartum cesarean delivery was associated with maternal age, nulliparity, previous cesarean, medical risk condition, preterm delivery, breech/transverse presentation, induced labor, and birth weight. Regardless of individual- and hospital-level characteristics, the higher the number of FTE obstetricians per 100 deliveries, the lower the intrapartum cesarean rate (aOR 0.55, 95% CI 0.36–0.83, *P*-value = 0.005), and the higher the number of FTE midwives per 100 deliveries, the lower the probability of elective cesarean delivery (aOR 0.79, 95% CI 0.69–0.90, *P*-value < 0.001).

**Table 3:** Multivariate analysis of factors associated with cesarean deliveries. Multilevel logistic regression models with hospital random effects.

	aOR [95% CI]		
	Urgent cesarean ^a^	Elective cesarean ^b^	Intrapartum cesarean ^C^
	(n = 2508/102 236)	(n = 10 243/99 728)	(n = 11 719/89 485)
Trend	0.95 (0.92–0.98)	1.00 (0.98–1.01)	1.01 (0.98–1.03)
**Women’s characteristics**			
Maternal age (years)	1.03 (1.02–1.04)	1.05 (1.05–1.06)	1.04 (1.03–1.04)
Nulliparous	1.55 (1.17–2.04)	1.09 (0.79–1.50)	5.02 (4.40–5.73)
Previous cesarean	5.09 (4.13–6.26)	25.22 (19.79–32.14)	10.99 (9.52–12.70)
Medical risk condition	1.82 (1.64–2.03)	1.42 (1.34–1.51)	1.19 (1.09–1.30)
Multiple pregnancy	0.40 (0.26–0.60)	1.00 (0.70–1.44)	0.82 (0.67–1.00)
Preterm delivery	4.46 (3.81–5.22)	0.77 (0.59–0.99)	1.29 (1.20–1.39)
Breech/transverse presentation	5.09 (4.01–6.45)	36.60 (28.16–47.57)	15.45 (11.78–20.26)
Induced labor	-	-	2.44 (2.30–2.57)
Birth weight (grams)			
< 2500	2.80 (2.38–3.30)	1.31 (1.02–1.67)	1.54 (1.35–1.76)
2500–4000	1	1	1
> 4000	0.92 (0.72–1.18)	1.56 (1.11–2.18)	2.02 (1.85–2.20)
**Maternity unit characteristics**			
Private	1.55 (1.00–2.40)	2.41 (1.41–4.15)	1.28 (0.86–1.89)
Teaching	0.76 (0.54–1.07)	1.29 (0.79–2.11)	0.98 (0.79–1.23)
Level of care			
No neonatology unit	1	1	1
Neonatology unit	1.11 (0.77–1.59)	1.37 (1.28–1.46)	0.96 (0.80–1.17)
Neonatal intensive care unit	1.08 (0.60–1.96)	1.83 (0.95–3.53)	0.96 (0.73–1.26)
Weekend/holiday delivery	1.12 (0.98–1.27)	0.12 (0.09–0.15)	0.96 (0.91–1.01)
On-call obstetrician outside the unit	0.87 (0.56–1.35)	0.90 (0.76–1.05)	1.07 (0.89–1.29)
Size (deliveries/year)			
< 1000	1.13 (0.95–1.35)	1.26 (1.16–1.36)	0.88 (0.76–1.03)
1000–1999	1	1	1
≥ 2000	1.18 (1.07–1.31)	0.95 (0.91–1.00)	1.06 (1.00–1.12)
Obstetricians (FTEs/100 deliveries)	1.26 (0.58–2.74)	0.91 (0.57–1.45)	0.55 (0.36–0.83)
Anesthesiologists (FTEs/100 deliveries)	1.33 (0.71–2.48)	0.99 (0.70–1.40)	1.14 (0.72–1.82)
Midwives (FTEs/100 deliveries)	1.40 (0.76–2.60)	0.79 (0.69–0.90)	1.11 (0.84–1.48)
**Between-hospital variance**	0.106 (0.033–0.336)	0.295 (0.200–0.436)	0.157 (0.094–0.264)
**Interclass correlation coefficient**	0.003 (0.000–0.033)	0.026 (0.012–0.055)	0.007 (0.003–0.021)
**Hosmer-Lemeshow test**	*P*-value = 1	*P*-value = 1	*P*-value = 1

aOR, adjusted odds ratio; CI, confidence interval; FTEs, full-time equivalents.

^a^ Urgent cesareans were compared with all other deliveries (elective cesareans, intrapartum cesareans, and vaginal deliveries).

^b^ Elective cesareans were compared with all deliveries with a trial of labor (intrapartum cesareans and vaginal deliveries).

^c^ Intrapartum cesareans were compared with all vaginal deliveries.

Because the obstetrician and midwife staffing levels were significantly associated with intrapartum and elective cesarean deliveries, respectively, we used an elasticity study to estimate the impact of a percentage change in these levels on the probability of a cesarean delivery. The elasticity for the probability of an intrapartum cesarean delivery with respect to the number of FTE obstetricians per 100 deliveries was -0.25 (95% CI -0.43,-0.08, *P*-value = 0.004). The elasticity for the probability of an elective cesarean delivery with respect to the number of FTE midwives per 100 deliveries was -0.34 (95% CI -0.52,-0.15, *P*-value < 0.001). The likelihood of an intrapartum cesarean delivery would have been associated with a decrease of 2.5 percentage points if the obstetrician level had increased by 10%, and that of an elective cesarean delivery with a decrease of 3.4 percentage points if the midwife level had increased by 10%. This means that intrapartum and elective cesarean delivery rates would have been associated with decreases of 19% (from 13.1% to 10.6%) and 33% (from 10.3% to 6.9%), respectively.

Rates of elective and intrapartum cesarean deliveries were stable while the use of prelabor urgent cesarean deliveries decreased over time. After adjustment for all the study factors, significant variability between hospitals remained for all types of cesarean deliveries (variance 0.106, 95% CI 0.033–0.336, for urgent cesarean deliveries; variance 0.295, 95% CI 0.200–0.436, for elective cesarean deliveries; and variance 0.157, 95% CI 0.094–0.264, for intrapartum cesarean deliveries). The non-significant *P*-values for the Hosmer-Lemeshow test validated the goodness-of-fit of the models.

Finally, we conducted sensitivity analyses. First, our analyses assumed that private part-time physicians worked 50% of a full-time equivalent. To check whether this staff weighting affected the results, we also considered two more extreme assumptions (25% and 75%). Our findings were similar both for the bivariate (S2 and S3 Tables) and the multivariate analyses (S4 and S5 Tables), regardless of the weighting used to assess the time private practice physicians spent in hospitals. Second, our multivariate models considered each hospital as a unit (random-effect models). To take into account correlations between maternity unit characteristics not included in the models and any independent variable, we re-estimated all models by considering each hospital as a variable (fixed-effect models). Results remained the same and thus confirmed that our findings are robust and conservative (S6–S8 Tables).

## Discussion

In this study, we observed a statistically significant association between staffing levels of the maternity unit and cesarean delivery use. Independently of all other characteristics, staffing levels for obstetricians and midwives had a significant impact on the use of intrapartum and elective cesarean deliveries, respectively.

As the number of FTE obstetricians per 100 deliveries per unit increased, the rate of intrapartum cesarean deliveries decreased. Several hypotheses might explain why higher obstetrician staffing levels in a maternity unit may be associated with a lower probability of intrapartum cesarean delivery. The presence of more obstetricians should improve the organization of obstetricians’ clinical practice. First, it might facilitate the availability of a full-time laborist in the unit. Recent studies report that a dedicated full-time laborist staff model is associated with lower cesarean delivery rates [54–56]. Second, obstetricians may share their decisions about mode of delivery more often with their colleagues who would be more available. Lessened time pressure might also encourage them to participate in their unit’s medical staff meetings and in the development of hospital-wide protocols for medical management, which in turn might improve their coordination. A Latin American cluster-randomized controlled trial found that hospitals applying a policy of mandatory second opinion, based on the best existing scientific evidence, reduced their cesarean delivery rates, mainly for intrapartum cesarean deliveries, without affecting maternal or perinatal morbidity or maternal satisfaction [57]. Likewise, a Canadian cluster-randomized controlled trial of a multifaceted intervention involving audits of indications for cesarean delivery, provision of feedback to health professionals, and implementation of best practices resulted in a significant reduction in the cesarean delivery rate, particularly for low-risk women, without adverse effects on maternal or neonatal outcomes [58]. Another study in the Netherlands examined the impact of the introduction of a cesarean delivery audit within the existing structure of daily report meetings in a regional teaching hospital. Implementing this obstetric audit facilitated the discussion among staff members about indications for cesarean delivery, improved awareness about lack of necessity of some cesarean deliveries, and thus decreased the hospital cesarean delivery rate [59]. An analysis in Taiwan examined physicians’ propensity for cesarean deliveries in obstetric clinics and found that physicians with solo practices were more likely to provide a cesarean delivery than those with group practices [60].

Similarly, as the number of FTE midwives per 100 deliveries per unit increased, the rate of elective cesarean deliveries decreased. The more midwives, the greater their potential participation in pregnancy care. Previous studies have showed that midwives may have a positive effect on outcomes of delivery, reducing the rates of some instrumental interventions, including cesarean deliveries [61–64]. Because midwives essentially provide care for low-risk women, they may be less affected by the fear of malpractice litigation, which numerous analyses have identified as a factor that increases the number of cesarean deliveries, particularly for obstetricians, who do manage high-risk pregnancies and may therefore be more likely to anticipate problems [43, 65, 66]. Accordingly, midwives may affect women’s preferences about labor and delivery, encouraging them to deliver normally [67–69]. Pregnant women are often anxious and afraid of delivery, which may lead them to feel uncertain about their ability to deliver vaginally [70, 71].

No staffing level was associated with urgent cesarean delivery. Nonetheless, this type of delivery accounts for only a small portion of all deliveries, 2.4% in our study, and is expected to be affected most strongly by medical risk factors.

The large size of our sample (more than 100 000 deliveries in 11 maternity units) as well as the high number of cesarean deliveries (more than 24 000) allowed us to obtain very accurate statistical estimates. The high quality of data is a major strength of our study. The CoNaissance 78 program dataset underwent a double quality control. The contents of the certificates were regularly checked by researchers at the General Council of Yvelines. The perinatal bureau of the Ile-de-France Regional Public Health Administration Agency performed a second consistency check under the scientific direction of Inserm Research Unit 1153. These quality control procedures prospectively enabled the correction of false and missing data, thus optimizing the accuracy and reliability of this dataset and the credibility of our results. Moreover, the information on hospitals extracted from the Annual Statistics for French Hospitals was checked and supplemented through the local perinatal network. Finally, we were able to disentangle the different types of cesarean deliveries. Since each of these types of cesarean delivery is performed under different conditions, this distinction between types is an added value of our study.

Our study nonetheless may have some limitations. Although we had access to a large set of individual- and hospital-level characteristics, confounding cannot be totally ruled out. We nonetheless controlled for many available covariates, which allowed us to reduce the potential for confounding. Body mass index (BMI) was not available but obesity, which is defined by a BMI ≥ 30 kg/m^2^, was available as a variable, so that we could adjust for it. Moreover, we could not adjust for ethnicity since this information was not available. The midwife staffing level variable was not significantly associated with elective cesarean delivery in the bivariate analysis, contrary to the multivariate analysis. Such a result was expected because staffing level variables are highly correlated with the characteristics of both patients and maternity units. This finding thus highlights the importance of the multivariate analysis.

Although our dataset was large, it concerned a single perinatal network of 11 hospitals. Nevertheless, both individual- and hospital-level characteristics in our study were similar to those in the 2010 National Perinatal Study, a representative population of 535 maternity units in France [11]. Similarly, the characteristics of the staff variables in the Yvelines district were almost the same as those nationwide [72]. Further analyses focusing on other regions or countries are desirable to confirm our results. Lastly, we assumed in our analyses that part-time private physicians worked 50% of their time at the hospital. This assumption may be a limitation. However, to ascertain that it did not affected our results, we also ran sensitivity analyses using two more extreme cases (25% and 75%) reporting similar results.

Our results may also have a broad public interest component outside France. Our models enabled us to take many staffing differences into account, according to hospital type and organization. All of these exist across healthcare systems. Therefore, our findings on medical staffing levels may be extended to other high-income countries, and in particular the results on midwife staffing to those with midwifery programs. In other healthcare settings where the staff of maternity units includes nurses instead of midwives, the potential impact of nursing staff on cesarean delivery rates should also be examined.

## Conclusion

Using a large database, we showed that levels of obstetricians and midwives affected cesarean delivery use. Decreasing unnecessary cesarean deliveries is clearly useful, but it is not yet clear how best to achieve this objective. Reassessment of the staffing levels of maternity units may be helpful since they are a potentially modifiable factor of the use of cesarean deliveries. High-quality research is still needed to evaluate the possible impact of changing staff configuration on perinatal outcomes.

## Supporting information

S1 Table Bivariate analysis of factors associated with cesarean deliveries. Multilevel logistic regression models with hospital random effects.
(DOCX)

S2 Table Bivariate analysis of factors associated with cesarean deliveries. **Multilevel logistic regression models with hospital random effects.** 25% extreme assumption for part-time private physicians.
(DOCX)

S3 Table Bivariate analysis of factors associated with cesarean deliveries. **Multilevel logistic regression models with hospital random effects.** 75% extreme assumption for part-time private physicians.
(DOCX)

S4 Table Multivariate analysis of factors associated with cesarean deliveries. **Multilevel logistic regression models with hospital random effects.** 25% extreme assumption for part-time private physicians.
(DOCX)

S5 Table Multivariate analysis of factors associated with cesarean deliveries. **Multilevel logistic regression models with hospital random effects.** 75% extreme assumption for part-time private physicians.
(DOCX)

S6 Table Multivariate analysis of factors associated with cesarean deliveries. **Multilevel logistic regression models with hospital fixed effects.** 50% assumption for part-time private physicians.
(DOCX)

S7 Table Multivariate analysis of factors associated with cesarean deliveries. **Multilevel logistic regression models with hospital fixed effects.** 25% extreme assumption for part-time private physicians.
(DOCX)

S8 Table Multivariate analysis of factors associated with cesarean deliveries. **Multilevel logistic regression models with hospital fixed effects.** 75% extreme assumption for part-time private physicians.
(DOCX)
