# Supplementary material for: Cesarean delivery rate and staffing levels of the maternity unit
Source: PLoS One. 2018 Nov 28;13(11):e0207379. doi: 10.1371/journal.pone.0207379 (PMC6261590; doi:10.1371/journal.pone.0207379)
Supplement: S3 Table — Multilevel logistic regression models with hospital random effects. 75% extreme assumption for part-time private physicians. (DOCX) [file pone.0207379.s003.docx]

**S3 Table. Bivariate analysis of factors associated with cesarean deliveries. Multilevel logistic regression models with hospital random effects.** 75% extreme assumption for part-time private physicians.

|  | **OR** [**95% CI**] | | |
| --- | --- | --- | --- |
|  | **Urgent cesarean ^a^** | **Elective cesarean** **^b^** | **Intrapartum cesarean** **^c^** |
|  | **(n = 2508/102 236)** | **(n = 10 243/99 728)** | **(n = 11 719/89 485)** |
| Obstetricians (FTEs/100 deliveries) | 1.14 (0.71-1.83) | 0.82 (0.64-1.05) | 0.59 (0.47-0.73) |
| Anesthesiologists (FTEs/100 deliveries) | 1.28 (0.64-2.55) | 1.00 (0.67-1.47) | 1.13 (0.73-1.73) |

OR, odds ratio; CI, confidence interval; FTEs, full-time equivalents.

^a^ Urgent cesareans were compared with all other deliveries (elective cesareans, intrapartum cesareans, and vaginal deliveries).

^b^ Elective cesareans were compared with all deliveries with a trial of labor (intrapartum cesareans and vaginal deliveries).

^c^  Intrapartum cesareans were compared with all vaginal deliveries.
