# Supplementary material for: Cesarean delivery rate and staffing levels of the maternity unit
Source: PLoS One. 2018 Nov 28;13(11):e0207379. doi: 10.1371/journal.pone.0207379 (PMC6261590; doi:10.1371/journal.pone.0207379)
Supplement: S5 Table — Multilevel logistic regression models with hospital random effects. 75% extreme assumption for part-time private physicians. (DOCX) [file pone.0207379.s005.docx]

**S5 Table. Multivariate analysis of factors associated with cesarean deliveries. Multilevel logistic regression models with hospital random effects.** 75% extreme assumption for part-time private physicians.

|  | **aOR** [**95% CI**] | | |
| --- | --- | --- | --- |
|  | **Urgent cesarean** **^a^** | **Elective cesarean** **^b^** | **Intrapartum cesarean** **^C^** |
|  | **(n = 2508/102 236)** | **(n = 10 243/99 728)** | **(n = 11 719/89 485)** |
| Trend | 0.95 (0.92-0.98) | 1.00 (0.98-1.01) | 1.00 (0.98-1.03) |
| **Women’s characteristics** |  |  |  |
| Maternal age (years) | 1.03 (1.02-1.04) | 1.05 (1.05-1.06) | 1.04 (1.03-1.04) |
| Nulliparous | 1.55 (1.17-2.04) | 1.09 (0.79-1.50) | 5.02 (4.40-5.73) |
| Previous cesarean | 5.09 (4.13-6.27) | 25.23 (19.79-32.15) | 10.99 (9.52-12.70) |
| Medical risk condition | 1.82 (1.64-2.03) | 1.42 (1.34-1.51) | 1.19 (1.09-1.30) |
| Multiple pregnancy | 0.40 (0.26-0.60) | 1.00 (0.70-1.44) | 0.82 (0.67-1.00) |
| Preterm delivery | 4.46 (3.81-5.22) | 0.77 (0.59-0.99) | 1.29 (1.20-1.39) |
| Breech/transverse presentation | 5.09 (4.01- 6.45) | 36.59 (28.16-47.56) | 15.45 (11.78-20.27) |
| Induced labor | - | - | 2.43 (2.30-2.57) |
| Birth weight (grams) |  |  |  |
| < 2500 | 2.80 (2.38-3.30) | 1.31 (1.02-1.67) | 1.54 (1.35-1.76) |
| 2500-4000 | 1 | 1 | 1 |
| > 4000 | 0.92 (0.72-1.18) | 1.56 (1.11-2.19) | 2.02 (1.85-2.20) |
| **Maternity unit characteristics** |  |  |  |
| Private | 1.56 (0.95-2.56) | 2.43 (1.42-4.17) | 1.29 (0.87-1.89) |
| Teaching | 0.76 (0.55-1.06) | 1.29 (0.79-2.11) | 0.98 (0.78-1.22) |
| Level of care |  |  |  |
| No neonatology unit | 1 | 1 | 1 |
| Neonatology unit | 1.10 (0.69-1.76) | 1.37 (1.28-1.47) | 0.98 (0.82-1.16) |
| Neonatal intensive care unit | 1.03 (0.54-1.95) | 1.81 (0.96-3.40) | 0.99 (0.76-1.27) |
| Weekend/holiday delivery | 1.12 (0.98- 1.27) | 0.12 (0.09-0.15) | 0.96 (0.91-1.01) |
| On-call obstetrician outside the unit | 0.84 (0.49-1.44) | 0.90 (0.76-1.05) | 1.08 (0.90-1.30) |
| Size (deliveries/year) |  |  |  |
| < 1000 | 1.16 (0.98-1.37) | 1.26 (1.18-1.35) | 0.88 (0.77-1.01) |
| 1000-1999 | 1 | 1 | 1 |
| ≥ 2000 | 1.18 (1.04-1.33) | 0.95 (0.91-0.99) | 1.06 (1.01-1.11) |
| Obstetricians (FTEs/100 deliveries) | 1.18 (0.62-2.26) | 0.89 (0.62-1.28) | 0.58 (0.41-0.82) |
| Anesthesiologists (FTEs/100 deliveries) | 1.20 (0.68-2.14) | 0.96 (0.70-1.31) | 1.16 (0.77-1.74) |
| Midwives (FTEs/100 deliveries) | 1.47 (0.73-2.96) | 0.79 (0.69-0.90) | 1.10 (0.83-1.48) |
| **Between-hospital variance** | 0.092 (0.014-0.596) | 0.294 (0.196-0.440) | 0.159 (0.095-0.268) |
| **Interclass correlation coefficient** | 0.003 (0.000-0.097) | 0.026 (0.012-0.056) | 0.008 (0.003-0.021) |
| **Hosmer-Lemeshow test** | *P*-value = 1 | *P*-value = 1 | *P*-value = 1 |

aOR, adjusted odds ratio; CI, confidence interval; FTEs, full-time equivalents.

^a^ Urgent cesareans were compared with all other deliveries (elective cesareans, intrapartum cesareans, and vaginal deliveries).

^b^ Elective cesareans were compared with all deliveries with a trial of labor (intrapartum cesareans and vaginal deliveries).

^c^ Intrapartum cesareans were compared with all vaginal deliveries.
