# Supplementary material for: Cesarean delivery rate and staffing levels of the maternity unit
Source: PLoS One. 2018 Nov 28;13(11):e0207379. doi: 10.1371/journal.pone.0207379 (PMC6261590; doi:10.1371/journal.pone.0207379)
Supplement: S6 Table — Multilevel logistic regression models with hospital fixed effects. 50% assumption for part-time private physicians. (DOCX) [file pone.0207379.s006.docx]

**S6 Table. Multivariate analysis of factors associated with cesarean deliveries. Multilevel logistic regression models with hospital fixed effects.** 50% assumption for part-time private physicians.

|  | **aOR** [**95% CI**] | | |
| --- | --- | --- | --- |
|  | **Urgent cesarean** **^a^** | **Elective cesarean** **^b^** | **Intrapartum cesarean** **^C^** |
|  | **(n = 2508/102 236)** | **(n = 10 243/99 728)** | **(n = 11 719/89 485)** |
| Trend | 0.96 (0.93-0.99) | 1.00 (0.98-1.02) | 1.01 (0.98-1.03) |
| **Women’s characteristics** |  |  |  |
| Maternal age (years) | 1.03 (1.02-1.04) | 1.05 (1.05-1.06) | 1.04 (1.03-1.04) |
| Nulliparous | 1.55 (1.17-2.05) | 1.09 (0.79-1.50) | 5.03 (4.40-5.75) |
| Previous cesarean | 5.08 (4.13-6.25) | 25.25 (19.80-32.19) | 11.00 (9.52-12.71) |
| Medical risk condition | 1.82 (1.63-2.03) | 1.42 (1.34-1.51) | 1.19 (1.09-1.30) |
| Multiple pregnancy | 0.40 (0.26-0.60) | 1.00 (0.69-1.44) | 0.82 (0.67-1.00) |
| Preterm delivery | 4.46 (3.81-5.22) | 0.77 (0.59-0.99) | 1.29 (1.20-1.39) |
| Breech/transverse presentation | 5.09 (4.02- 6.44) | 36.63 (28.16-47.65) | 15.47 (11.79-20.29) |
| Induced labor | - | - | 2.44 (2.31-2.58) |
| Birth weight (grams) |  |  |  |
| < 2500 | 2.80 (2.38-3.30) | 1.31 (1.03-1.67) | 1.54 (1.35-1.76) |
| 2500-4000 | 1 | 1 | 1 |
| > 4000 | 0.92 (0.72-1.18) | 1.56 (1.11-2.18) | 2.02 (1.85-2.20) |
| **Maternity unit characteristics ^d^** |  |  |  |
| Weekend/holiday delivery | 1.12 (0.98- 1.27) | 0.12 (0.09-0.15) | 0.96 (0.91-1.01) |
| On-call obstetrician outside the unit | 1.31 (0.91-1.88) | 1.16 (1.01-1.34) | 1.12 (0.90-1.39) |
| Size (deliveries/year) |  |  |  |
| < 1000 | 1.06 (0.88-1.27) | 1.21 (1.10-1.32) | 0.89 (0.81-0.98) |
| 1000-1999 | 1 | 1 | 1 |
| ≥ 2000 | 1.21 (1.06-1.37) | 1.00 (0.93-1.07) | 1.04 (1.00-1.09) |
| Obstetricians (FTEs/100 deliveries) | 1.46 (0.62-3.43) | 0.93 (0.56-1.54) | 0.61 (0.43-0.87) |
| Anesthesiologists (FTEs/100 deliveries) | 1.47 (0.66-3.27) | 1.04 (0.68-1.61) | 1.07 (0.68-1.67) |
| Midwives (FTEs/100 deliveries) | 1.05 (0.65-1.68) | 0.78 (0.64-0.95) | 1.08 (0.82-1.42) |
| **Hosmer-Lemeshow test** | *P*-value = 1 | *P*-value = 1 | *P*-value = 1 |

aOR, adjusted odds ratio; CI, confidence interval; FTEs, full-time equivalents.

^a^ Urgent cesareans were compared with all other deliveries (elective cesareans, intrapartum cesareans, and vaginal deliveries).

^b^ Elective cesareans were compared with all deliveries with a trial of labor (intrapartum cesareans and vaginal deliveries).

^c^ Intrapartum cesareans were compared with all vaginal deliveries.

^d^ Hospital invariant variables (private, teaching, level of care) were not included in the models because of the strict collinearity with hospital fixed effects. However, using hospital fixed effects models, we controlled by all hospital characteristics.
